# Translational research approach to social orienting deficits in autism: the role of superior colliculus-ventral tegmental pathway

**DOI:** 10.1038/s41380-025-02962-w

**Published:** 2025-04-05

**Authors:** Alessandro Contestabile, Nada Kojovic, Giulia Casarotto, Farnaz Delavari, Patric Hagmann, Marie Schaer, Camilla Bellone

**Affiliations:** 1 Department of Basic Neuroscience, Faculty of Medicine, University of Geneva, Geneva, Switzerland; 2 Department of Psychiatry, Faculty of Medicine, Geneva University Hospitals, Geneva, Switzerland; 3 Department of Radiology, University Hospital of Lausanne and University of Lausanne, Lausanne (CHUV-UNIL), Vaud, Switzerland; 4 Present Address: Department of Neuroscience, Karolinska Institutet, Biomedicum, Stockholm, Sweden

**Keywords:** Neuroscience, Autism spectrum disorders

## Abstract

Autism Spectrum Disorder (ASD) is characterized by impairments in social interaction and repetitive behaviors. A key characteristic of ASD is a decreased interest in social interactions, which affects individuals’ ability to engage with their social environment. This study explores the neurobiological basis of these social deficits, focusing on the pathway between the Superior Colliculus (SC) and the Ventral Tegmental Area (VTA). Adopting a translational approach, our research used Shank3 knockout mice (*Shank3*^*−/*−^), which parallel a clinical cohort of young children with ASD, to investigate these mechanisms. We observed consistent deficits in social orienting across species. In children with ASD, fMRI analyses revealed a significant decrease in connectivity between the SC and VTA. Additionally, using miniscopes in mice, we identified a reduction in the frequency of calcium transients in SC neurons projecting to the VTA, accompanied by changes in neuronal correlation and intrinsic cellular properties. Notably, the interneuronal correlation in *Shank3*^*−/*−^ mice and the functional connectivity of the SC to VTA pathway in children with ASD correlated with the severity of social deficits. Our findings underscore the potential of the SC-VTA pathway as a biomarker for ASD and open new avenues for therapeutic interventions, highlighting the importance of early detection and targeted treatment strategies.

## Introduction

Autism Spectrum Disorder (ASD) is a neurodevelopmental disorder characterized by a diverse range of symptoms, including impairments in social communication and interaction, alongside restricted and repetitive behaviors [1]. Diminished social interest significantly impacts the ability to engage with others, and while the origins of these deficits are widely debated, deficits in social motivation are frequently highlighted. However, the neurobiological mechanisms underpinning the social motivation hypothesis are still unclear.

Social motivation is a set of psychological dispositions and biological mechanisms that bias individuals to preferentially orient themselves to the social world, seek and take pleasure in social interactions, and work to foster and maintain social bonds [2, 3]. The theory posits that individuals with ASD present deficits in social motivation, which manifest in decreased attention to social information [4] and lower reward levels from social interactions. Despite challenges, this theory has shaped research on social-orienting deficits in ASD, underscoring the importance of early detection and intervention to improve outcomes for children with developmental delays [5, 6].

Eye-tracking methodologies have provided empirical evidence to elucidate the social motivation hypothesis [7, 8] and have contributed to the quantitative and qualitative analysis of the alterations in social attention in autism [4, 9, 10]. Eye-tracking studies on toddlers with ASD revealed a marked reduction in their orientation to social information based on simple visual preference paradigms [11, 12]. In terms of qualitative differences, when viewing complex social scenes, individuals with ASD exhibit a higher degree of gaze idiosyncrasy compared to the typically developing population [13, 14]. One striking example, as highlighted by studies using face stimuli, is that individuals with ASD tend to focus on the lower part of the face rather than the eye region, which is a highly dynamic area known to convey intentions and emotional states [9, 15]. Impairments in social visual engagement are considered as the main pathognomic feature in ASD arising from the earliest months of life [16]. Recent studies have indicated that individuals with ASD often exhibit atypical attentional mechanisms. For example, early in development individuals with ASD show difficulties in shifting their attention [17], a phenomenon that is associated with the emergence of core ASD symptoms [18]. The evidence from the eye-tracking studies strongly supports the need for early interventions that target social orienting as a key mechanism for enhancing the developmental outcomes of the children with the largest developmental delays. Despite these findings, the neural circuits underlying social orienting deficits in ASD remain largely unexplored. Johnson [19] postulated that the abnormalities in the processing of face information that characterize autism may have their roots in phylogenetically primitive brain regions. Specifically, they proposed that the disruption in the subcortical automatic face processing route involving the Superior Colliculus (SC)-Pulvinar (PUL) and Amygdala (AMY) contributes to autism’s social orienting deficits. This pathway is involved in the rapid, automatic processing of face stimuli [19, 20]. Early disturbances in the functioning of this circuit might lead to the aberrant social orienting and downstream influence maturation of the cortical regions involved in the processing of social information.

The SC is a layer structure located in the brainstem and processes incoming visual stimuli based on their significance and importance to orienting behaviors [21]. Non-invasive human imaging studies have reported SC activation during selective attention tasks [22], and specific neuronal populations within the SC have been implicated in attentional disengagement processes [23]. In rodents, it has been demonstrated that the intermediate and deep layers of the SC form synaptic contacts with both dopaminergic (DA) and non-DA neurons in the substantia nigra pars compacta (SNc) and the Ventral Tegmental Area (VTA), providing rapid visual inputs [24, 25]. These inputs are implicated in reinforcement learning [26] and play a pivotal role in conspecific interaction [25]. Despite these findings, the role and the neuronal mechanisms underlying the role of SC in social deficits in ASD are unknown.

In our study, we employ a translational approach using *Shank3* knockout mice as an animal model related to ASD, alongside a clinical cohort of young children with ASD (aged 1.6–4.4 years). Our findings reveal that both mice and children exhibit deficits in social orienting behavior. In human participants, we observed diminished connectivity between the SC and VTA using seed-based spontaneous sleep functional connectivity (fMRI). To further investigate the neuronal mechanisms underlying social orienting and the role of SC to VTA pathway, we employed miniscopes and observed a decrease in the frequency of calcium transients in SC neurons projecting to the VTA. These deficits were accompanied by alterations in interneuronal correlations of calcium activity and intrinsic cell properties. Finally, our data demonstrate a correlation between these neuronal deficits and orienting behaviors in humans and mice, suggesting that SC-VTA activity may serve as a viable biomarker for stratifying autistic children for therapeutic interventions.

## Materials and methods

### Animal research

#### Mice

The experimental procedures described here were conducted in accordance with the Swiss laws and previously approved by the Geneva Cantonal Veterinary Authority. C57Bl/6j Shank3Δe4-22 male and female mice (also described as *Shank3*^*+/+*^ and *Shank3*^*−/−*^) were obtained from Yong-hui Jiang laboratory [27] and bred in our animal facility. Naive juvenile (3–4 weeks old) male and female C57Bl/6j were used as stimuli animals in the social orientation test and free social interaction. All C57Bl/6j mice were group-housed (2–5 per cage) in the institutional animal facility under standard 12/12 h light/dark cycles with food and water *ad libitum*. Young mice were weaned and separated by gender at P21. Behavioral experiments were conducted in a room with fixed low illumination (10–15 lux) and with controlled humidity (40%) and temperature (22–24 °C). The experiments were always performed within a time frame that started approximately 2 h after the end of the dark circle and ended 2 h before the start of the next dark circle.

#### Viruses

AAVrg-Ef1α-mCherry-IRES-Cre (titer ≥7 × 10^12^ vg/mL, Addgene), ssAAV-1/2 hSyn1-dlox-jGCaMP7f(rev)-dlox-WPRE (v319-1, titer = 3.8 × 10^12^ vg/mL, Viral vector ETH Zurich), AAV9-hEF1α-dlox-EGFP-dlox-WPRE (v544-9, titer = 3.8 × 10^12^ vg/mL, Viral vector ETH Zurich).

#### Social behavior tests

The arena of the orientation test consisted of two cylinders (height = 25 cm) positioned one inside the other. The smaller cylinder (∅ = 8 cm) was composed of transparent plastic and presented small holes (∅ = 0.3 cm) which prevented social contact but allowed olfactory, auditory and visual cues. The big cylinder (∅ = 20 cm) instead was composed by opaque plastic. This test protocol was extensively described in [25]. Briefly, the experimental mice were gently placed in the internal small cylinder, which allowed the experimental animal only to rotate in the left or right direction. After a 5 min period of habituation, a social stimulus (unfamiliar juvenile conspecific sex matched C57BL/6J) was introduced in the circular corridor formed by the small and big cylinder. The stimulus was allowed to freely move in the inter-cylinder space for 5 min. The arena was cleaned using 70% ethanol between each trial.

Similarly to [25, 28], the free social interaction was performed in an arena with the same dimension of the home cage (35 × 20 × 15 cm). Each mouse was placed individually in the center of the arena and recorded during a habituation (5 min) and a consecutive free interaction period (5 min). Between each trail, the apparatus was cleaned with 70% alcohol.

Every session of social behavior tests was video-tracked and recorded using Ethovision XT (Noldus, Wageningen, the Netherlands). Using the tracking of the body parts - the gravity center and the nose – it was possible to establish the relative position in space, the head orientation angle and consequently the time passed with the social stimulus in the frontal field (head-oriented angle <45° per side). Manual scoring was performed to quantify the time passed sniffing the social stimulus.

#### Surgery on mice

Stereotaxic injections: Mice were anesthetized with a mixture of oxygen (1 L/min) and isoflurane 3% (Baxter AG, Vienna, Austria). The skin was shaved, locally anesthetized with 40–50 μL lidocaine 0.5% and disinfected. The animals were placed in a stereotactic frame (Angle One; Leica, Germany) and the virus was injected in the brain. In GCaMP recorded experiments, AAVrg-Ef1α-mCherry-IRES-Cre was unilaterally injected in the VTA (AP: −3.2 mm, ML: + or −0.5, DV: −4.25/−4.0 mm from Bregma, 400 nL in total) and AAV9-hSyn-FLEX-GCaMP7f-WPRE-SV40 was unilaterally injected in the SC (AP: −3.4 mm, ML: + or −0.8, DV: −1.5 mm from Bregma, 500 nL). The viruses were incubated 3–4 weeks prior to miniscope implantation.

GRIN lens implantation: As described in the paragraph above, the animals were anesthetized, placed in a stereotactic frame and the skin was shaved. After scratching the skull surface, dental adhesive resin cement was applied on the skull (Super-Bond kit, SUN MEDICAL CO., LTD, Shiga; Japan) and a unilateral craniotomy was performed above the SC (1.2 mm^2^ cranial window centred above the SC (AP: −3.4 mm, ML: +0.8 mm). Afterwards, a gradient-index (GRIN) lens (Inscopix ©, diameter: 1 mm, length: 4 mm) was implanted (DV: −1.5 mm from Bregma) above the SC and fixed to the skull with Super-Bond and dental acrylic. After surgery the mice were followed each day until the behavioral experiment (3–5 weeks after surgery).

Validation: After the behavioral experiments the animals were sacrificed by lethal injection of pentobarbital and transcardially perfused with phosphate buffer solution (PBS) 1× followed by 4% paraformaldehyde prepared in PBS 1x. The brain was then extracted and stored at 4 °C overnight to postfixate in the same solution. The ROIs of the brain were sliced in 50 μm thick coronal slices (Leica VT1000 S). Finally, slices were mounted using Fluoroshield mounting medium with DAPI (abcam, Cat#ab104139) and imaged using a widefield Axio Scan.Z1 scanner.

#### Miniscope experiment and data analysis

During free social interaction (see protocol above), calcium recordings of single SC-VTA neurons were performed using an nVista miniaturized epi-fluorescent microscope (Inscopix ©). All recordings were made at 10 frames per second. Optimal field of view was determined the day before the test and was controlled during habituation. LED intensities (0.1–2 mW) and gain (1–5×) were optimized per animal for sufficient signal-to-noise ratio and for minimizing fluorescent bleaching. Timing of each acquired frame (SYNC-port on Inscopix data acquisition box) was synchronized with behavioral recordings through TTL signal. The acquired tiff videos were spatially downsampled (4×), cropped, and motion corrected using Inscopix data processing software. Identification of regions of interest (ROI) – in this case, SC-VTA GCaMP positive neurons – was performed using a constrained non-negative matrix factorization approach extended for microendoscopic data (CNMF-E). CNMF-E algorithms (custom-written MATLAB codes) published by [29] were used to automatically remove potential noise ROIs. To further improve the quality of the isolation and to avoid double count, manual quality control was performed by inspecting the spatial and temporal data for each ROI. After calculation of z-score, ROIs with poor signal/noise quality were also excluded. Convolution was applied on recorded signals. The partial correlation networks and the relative correlations between neuronal activity were calculated only for the brains with at least 8 recorded neurons and were estimated using the EBIC and the Lasso algorithm (hyperparameter = 0.5; [30]. These codes are part of *qgraph* and *glasso* packages in R. The construction of peri-event time histogram (PETH) was made by aligning specific behavioural events (ipsilateral and contralateral orientations) to the neuronal activity of each neuron. These events were identified by manual scoring or through body parts tracking. Area under the curve (AUC) from mean z-scored signals were assessed from −2–0 s and from 0–2 s. The difference of AUC (ΔAUC = AUC^[0; 2]^−AUC^[−2; 0]^) was calculated to quantify the percentage of responding neurons to an episode.

#### Whole-cell patch clamp recordings

Coronal midbrain slices 250 μm thick containing the SC were prepared following the experimental injection protocols described above. Brains were sliced by using a cutting solution containing: 90.89 mM choline chloride, 24.98 mM glucose, 25 mM NaHCO3, 6.98 mM MgCl2, 11.85 mM ascorbic acid, 3.09 mM sodium pyruvate, 2.49 mM KCl, 1.25 mM NaH2PO4, and 0.50 mM CaCl2. Brain slices were incubated in cutting solution for 20–30 min at 35°. Subsequently, slices were transferred in artificial cerebrospinal fluid (aCSF) containing: 119 mM NaCl, 2.5 mM KCl, 1.3 mM MgCl2, 2.5 mM CaCl2, 1.0 mM NaH2PO4, 26.2 mM NaHCO3, and 11 mM glucose, bubbled with 95% O2 and 5% CO2) at room temperature. Whole-cell voltage-clamp or current-clamp electrophysiological recordings were conducted at 32–34° in aCSF (2–3 ml/min, submerged slices). Recording pipette contained the following internal solution: 140 mM K-Gluconate, 2 mM MgCl2, 5 mM KCl, 0.2 mM EGTA, 10 mM HEPES, 4 mM Na2ATP, 0.3 mM Na3GTP, and 10 mM creatine-phosphate. The cells were recorded at the access resistance from 10–30 MΩ. Resting membrane potential (Vm in mV) and cell capacitance (Cp in pF) were measured in voltage-clamp at −60 mV using the Multiclamp 700B Commander (Molecular Devices) while injecting no current (I = 0) immediately after breaking into a cell. In the current clamp recording, the current was injected in ramp mode (0–300 pA for 1 s) for measuring the firing threshold and rheobase. Action potentials (APs) were elicited in current-clamp configuration by injecting depolarizing current steps (50 pA, 500 ms) from 0–400 pA. After-hyperpolarization current (AHP) was assessed in voltage clamp configuration by holding the cell at −60 mV with a step of +60 mV for 100 ms. Whole-cell patch clamp recordings were performed without and in presence of Picrotoxin (100 µM) and Kynurenic acid (3 mM).

### Human research

#### Ethics approval and consent to participate

Data for the current study were acquired as a part of a larger longitudinal study of early behavioral and brain development in autism based in Geneva. Detailed information about the cohort is given in more detail elsewhere [31]. This study protocol was approved by the Ethics Committee of the Faculty of Medicine of Geneva University, Switzerland (12-163/Psy 12-014, referral number PB_2016-01880). Written informed consent was obtained from all parents or their legal guardians of children prior to their participation in the study. All procedures in this study were performed in accordance with relevant institutional and national guidelines and regulations, as well as the Declaration of Helsinki, ensuring adherence to ethical standards for research involving human participants.

#### Human sample

Forty-six children with autism (10 girls) (aged 2.80 ± 0.77 years) participated in the study. Table 1 summarizes the clinical characteristics of the human sample. Brain images were acquired exclusively with ASD participants, as the recruitment process did not yield a sufficient number of TD volunteers for MRI procedures. All children presented a clinical autism diagnosis according to the DSM-5 criteria which relied both on the standardized observational assessment of the child and interviews with caregivers(s) retracing the child’s medical and developmental history. All children with ASD reached the cut-off for ASD on Autism Diagnostic Observation Schedule-2 [32].

**Table 1:** Behavioral characteristics of the human sample.

Behavioral measures	ASD (*n* = 46) Mean ± SD
Age	2.80 ± 0.77
Total Symptom Severity Score (ADOS-2 CSS)	7.44 ± 1.77
Social Affect (ADOS-2 SA-CSS)	6.40 ± 1.90
Repetitive Behaviors & Restricted Interests (ADOS-2 RRB CSS)	9.07 ± 1.19
MSEL Early Learning Composite DQ	68.08 ± 23.89
MSEL Visual reception DQ	80.54 ± 25.38
MSEL Fine Motor DQ	82.49 ± 20.39
MSEL Expressive language DQ	56.65 ± 31.99
MSEL Receptive language DQ	52.64 ± 29.17

To robustly characterize the behavioral functioning of the included ASD group relative to a typically developing (TD) population we created 100 simulated TD samples drawn from our larger longitudinal behavioral cohort in Geneva [31]. These simulated samples were matched to our ASD group in terms of size, age, and gender composition. We opted for the iterative comparisons with simulated TD samples instead of simple group comparison to achieve the appreciation of variability and confidence in the differences observed between the ASD and TD groups.

#### Behavioral phenotype measures

Autistic symptoms were measured using Autism Diagnostic Observation Schedule-2nd edition (ADOS-2) [32]. The ADOS provides a global measure of the severity of autistic symptoms that ranges from 1–10 [33, 34], as well as a more precise measure of symptoms according to their type, namely, social affect (SA) and restricted and repetitive behaviors (RRB) [35].

Developmental functioning in children was measured using Mullen Early Learning Scales -MSEL [36]. The composite measure of the development functioning, the Early Learning Composite Score, was obtained by averaging the performance in the four “cognitive” domains: visual reception, fine motor, receptive, and expressive language. The MSEL presents a strong floor effect, with t-scores per scale starting at 20. To accommodate lower-functioning children in our sample and avoid this floor effect, we employed the developmental quotient (DQ). The DQ is obtained by dividing the age equivalent (AE) scores by the chronological age (CA) of the child.

#### Eye-tracking derived measures of social orientation

Gaze data for both eye tracking tasks were collected using Tobii TX300 eye tracker (https://www.tobiipro.com), sampled at 300 Hz. Participants were positioned approximately 60 cm from the recording screen (1200 × 1920 pixels, refresh rate of 60 Hz). Calibration was carried out using a five-point procedure featuring child-friendly animations within the Tobii system’s built-in program. If the eye-tracking device did not accurately register the participant’s gaze position, the calibration was redone. The testing environment was controlled for consistency in lighting conditions, as the room had no windows.

#### Visual preference

To assess preference for social information (SOC), we used an eye-tracking task [12] inspired by the task of Pierce et al. [11].This task displayed dynamic geometric motion GEO and SOC videos in a split-screen format simultaneously for one minute. The GEO component involved abstract shapes reminiscent of classic screensavers, while the SOC component featured videos of children dancing against a neutral background.

Data was analyzed using Tobii Studio software (version 3.4.8), measuring the fixation duration on SOC and GEO. We calculated fixation duration on each stimulus and measure of social orienting is obtained by dividing the time spent viewing the social information divided by the total time viewing the stimulus.

#### Complex social scene

The measure of the typicality of the gazing pattern, compared to a normative group of typically developed children, was obtained using the procedure explained in our previous work [14]. The current experiment consisted of free viewing of one episode of the French cartoon “Trotro” lasting 2′53″ [37]. The cartoon depicts a simple social plot and appeals to young children.

The normative gaze distribution (norm) was obtained by calculating the kernel density distribution estimation function on gaze data from TD individuals on each frame of the video. We used TD recordings acquired in the context of a larger longitudinal cohort [31]. To assess the stability of the observed differences, we generated 100 normative samples that closely matched the characteristics of the children in the ASD group with eye-tracking data for the specified task in terms of number, sex, and age. In our ASD sample the eye-tracking was acquired in 38 out of 46 children (9 girls); aged 2.90 ± 0.74 years). Upon the “norm” definition, we calculated the distance of gaze data from this norm on each frame for each child with ASD. This measure of distance to the normative gaze distribution, Proximity Index-PI), ranges from 0 (outside of the focus of attention defined by 38 TD children) to 1 (gaze coordinates coincide with the very center of the attentional distribution of the TD children on the given frame).

#### MRI acquisition in children

MRI images were acquired on a 3T Siemens Magnetom Prisma (Siemens Medical Solutions, Erlangen, Germany) using a 64-channel head coil at Campus Biotech, Geneva, Switzerland. The imaging was performed in children’s natural sleep at night time, without sedation, following a modified procedure from Nordahl and collaborators [38]. A comprehensive sleep history was obtained for all participating children to develop a personalized MRI recording strategy. Prior to the imaging session, the children underwent behavioral training at home under the supervision of the psychologist (NK) to help them adapt to the novel environment and tolerate the noise of the MRI machine while asleep.

#### Functional image acquisition and preprocessing

Functional scans in nocturnal sleep were acquired using a T2-weighted sequence (700–800 frames, FoV = 224 mm, voxel size = 2.4 × 2.4 × 2.6 mm, 48 axial slices, TR = 500 ms, TE = 33 ms, flip angle = 47°, phase encoding anterior > posterior, interleaved) for a minimum of 6 min. Functional images were preprocessed using SPM12 (Wellcome Trust Centre for Neuroimaging, London, UK; http://www.fil.ion.ucl.ac.uk/spm) and an in-lab pipeline (described in [39]) using functions of the DPARSF [40] and IBASPM [41] toolboxes.

#### Region of interest definition and connectivity

The definition of the superior colliculus (SC) region of interest (ROI) was done using the FSL’s Juelich histological atlas [42–44]. Evidence from fetal postmortem research indicates that human SC and its connections are fully mature by the mid-gestation [45]. Thus, the use of the adult brain atlas was deemed appropriate. The ventral tegmental area (VTA) was defined using the probabilistic VTA atlas based on in vivo 7T MRI multimodal data [46].

Using the study specific DARTEL template [47] the ROIs were spatially transformed into the individual subject space. A bilateral mask was created for all ROIs, and for the case of VTA where a probabilistic atlas was used, we included voxels with a probability of belonging to the respective ROI higher than 35%. To minimize the effects of the interindividual anatomic variability and following others [48] each voxel’s time series was weighted by the probability of inclusion in the ROI. This was done to ensure that voxels most reliably located in each region made the most significant contribution to its signal. For each subject, ROI functional connectivity maps in the individual subject-space were created by computing the Pearson correlation between each voxel’s time-course and the weighted average of the time course of the [48].

### Statistical analyses

Statistical analysis of rodent related data was conducted with GraphPad Prism 8 (San Diego, CA, USA) or R studio system. The normality of sample distributions was assessed with the Shapiro–Wilk criterion and when violated, non-parametric statistics were applied (Mann-Whitney for two groups comparison, while for multiple comparisons, Kruskal–Wallis or Friedman tests were followed by Dunn’s test). When samples were normally distributed, data were analysed with independent or paired two-tailed sample t-tests, one-way, two-way, or repeated measures analysis of variance (ANOVA) followed if significant by Bonferroni post hoc tests. All error bars represent the mean ± SEM and the significance was set at p < 0.05.

Statistical analyses for human data were performed using MATLAB (version 2021a; MathWorks, Natick, MA). Group differences were assessed with independent two-sample t-tests. Multivariate association patterns between the SC to whole-brain functional connectivity and behavioral variables were examined using Partial Least Squares Correlation (PLS-C), see Supplementary Information. For univariate associations between the SC-VTA (ROI to ROI) and behavioral variables, both Pearson and Spearman correlation coefficients were calculated, as appropriate. To identify and remove outliers in the correlation analyses, a median absolute deviation (MAD) threshold of 3 was employed. The exclusion of outliers did not alter the statistical significance of the correlational findings.

## Results

### ASD-related mouse model and autistic children present an impairment in social orientation

**Fig. 1:**
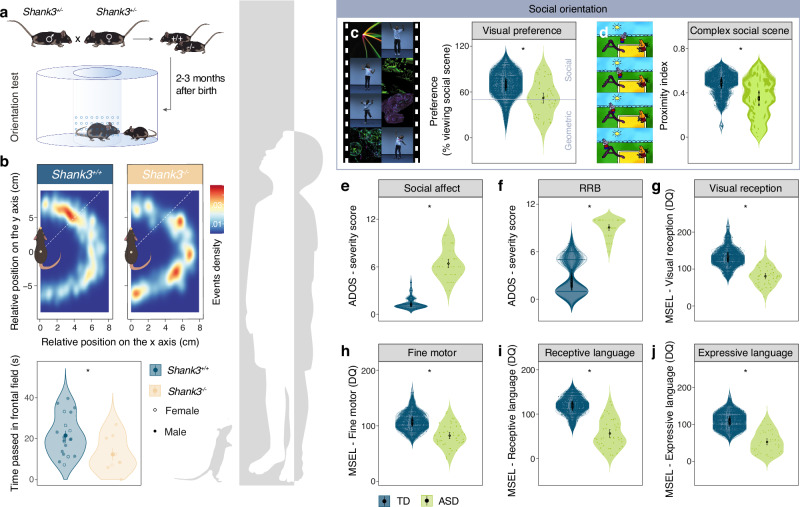
Shank3 KO mice and autistic children show deficits in social orientation. **a** Schematic representation of the breeding and the social orientation test. **b** Upper panel: heatmaps reporting the relative position of the social stimulus during orientation test. Lower panel: violin plots reporting the time passed with the juvenile stimulus in the frontal field displayed for *Shank3*^*+/+*^ (n = 20) and *Shank3*^*−/−*^ (n = 9) mice (unpaired t-test: p-value = 0.034). The mean and s.e.m. are indicated per group. Human sample (panels **c**–**j**) demonstrate group comparisons obtained by using t-test comparing the ASD sample (green) to 100 bootstrapped typically developing (TD) samples (blue). **c** Left panel: example frames of the video used for the experiment. Right panel: violin plots reporting the eye-tracking derived visual preference for social information (TD, n = 46) and ASD (n = 46) children (p-value < 0.001, median cohen D = 0.95). **d** Left panel: example frames of the video used for the experiment. Right panel: violin plots reporting proximity index derived from viewing a complex social scene TD (n = 38) and ASD (n = 38) children (p-value < 0.001, median cohen D = 1.04). **e** Symptom severity in social affect domain of Autism Diagnosis Observation Schedule (ADOS) for TD (n = 46) and ASD (n = 46) children (p-value < 0.001, median cohen D = −3.45). **f** Symptom severity in restricted and Repetitive Behaviors (RRB) domain of ADOS for TD (n = 46) and ASD (n = 46) children (p-value < 0.001, median cohen D = −4.25). Mullen Early Learning scales (MSEL) Developmental quotient (DQ) for TD (n = 46) and ASD (n = 46) children in **g** Visual reception (p-value < 0.001, median cohen D = 2.04); **h** Fine Motor (p-value < 0.001, median cohen D = 1.32); **i** Receptive Language (p-value < 0.001, median cohen D = 2.39); and (**j**) Expressive Language (p-value < 0.001, median cohen D = 2.21).

We employed a previously characterized orientation task [25] to investigate social orienting responses in Shank3 Knock-out (*Shank3*^*−/−*^) mice, a well-established ASD-related mouse model which presents impairments in social interaction [27, 28] and social preference [49]. The experimental mouse was introduced and restrained in an enclosure positioned in the center of a circular arena, allowing it to turn right or left. After 5 min habituation, we placed a sex-matched juvenile conspecific in the arena, and we calculated the orienting response toward the conspecific (Fig. 1a). Compared to littermate controls (*Shank3*^*+/+*^), we observed that *Shank3*^*−/−*^ mice spent less time with the conspecific social stimulus in their respective frontal field (Fig. 1b), indicating a reduced social orientation.

Parallel studies in rodents and humans offer a unique opportunity to understand the complexity of social deficits in ASD and to reveal neuronal mechanisms underlying them. We compared the behavioral characteristics, social orientation, autistic symptoms, and developmental functioning of a human sample of ASD children with those of typically developing children (TD) participating in an ongoing longitudinal study in Geneva [31]. For assessing social orientation, we employed two established paradigms [12, 14]. In the first controlled visual preference paradigm [12], we quantified the time children spent observing social stimuli compared to simultaneously presented geometric stimuli. Children in the ASD group exhibited a markedly reduced interest in social content presented on the screen (Fig. 1c). In the second paradigm, we presented a more complex social scene, where we compared the gaze patterns of children with ASD to the reference gaze distribution of TD children using an in-house developed methodology [14]. This comparison was conducted while both groups watched a 3-min cartoon. We employed a data-driven approach to quantify the divergence in gaze patterns between children with ASD and TD children. Our analysis showed that children with ASD demonstrated gaze patterns that significantly diverged from those of TD children, (Fig. 1d). To obtain a standardized measure of autistic symptoms we used the ADOS-2 (Autism Diagnostic Observation Schedule, Second Edition) [32]. In accordance with their diagnostic classification, children with ASD exhibited a significantly higher presence of symptoms in both the social affect (SA) and the domain of restricted and repetitive interests (RRB) (Fig. 1e, f). Finally, compared to TD children, children with ASD demonstrated greater challenges in several key developmental areas assessed using Mullen Early Learning Scales (MSEL, Mullen [36]): Visual reception, Fine motor skills, and both Receptive and Expressive language (Fig. 1g–j).

### MRI revealed a lower connectivity between SC, VTA and other brain areas in children with ASD

**Fig. 2:**
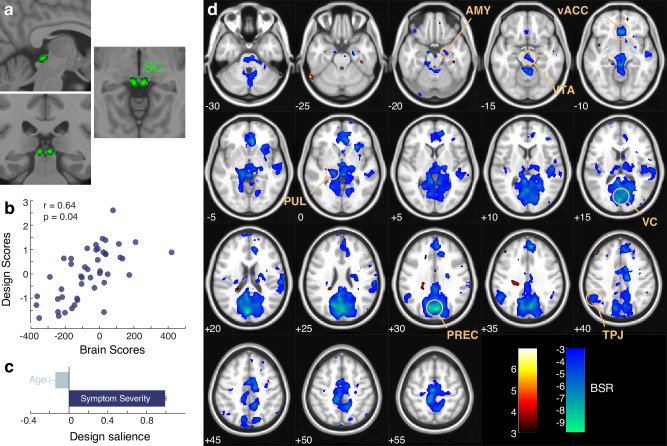
Partial least squares correlation (PLS-C) analysis of superior colliculus (SC) whole-brain functional connectivity with age and symptom severity in 46 children with ASD. **a** Selection of the superior colliculus (SC) as region of interest (ROI). **b** Scatter plot depicting correlation (Pearson) between brain (x-axis) and design (y axis) scores. **c** The design saliences from the significant latent variable (LV) demonstrate a pronounced positive effect of the level of autistic symptoms and a negligible negative effect of age. **d** The brain salience pattern reveals regions of decreased connectivity with the SC (blue, negative bootstrap ratio (BSR)) in children exhibiting higher symptom severity. Or more severe ASD symptoms correlate with reduced connectivity between the SC and regions integral to attention and reward processing, including the ventral tegmental area (VTA), pulvinar (PUL), ventral anterior cingulate cortex (vACC), precuneus (PREC), primary visual cortex (VIS), left amygdala (AMY), and vermis (VERM).

Using the **superior colliculus (SC)** as the seed region (Fig. 2a), we assessed the multivariate pattern of seed-to-whole brain functional connectivity and its association with age and autistic symptom severity, by applying the partial least squares correlation (PLS-C) [50, 51]. PLS-C revealed one significant component (r = 0.64, p = 0.04, Fig. 2b). This component (as depicted in Fig. 2c) showed the main (positive) effect of autistic symptomatology, while the (negative) effect of age was less important. The multivariate brain pattern indicated that in children with ASD, higher levels of symptoms co-occurred with hypoconnectivity between the SC and regions involved in reward processing and attention (clusters shown in blue in Fig. 2d). Namely, in the context of the enhanced symptoms, we found a pattern of lower connectivity between the SC and VTA (bilateral), pulvinar (bilateral), right amygdala, visual cortex (bilateral), precuneus (bilateral), vermis and left temporoparietal junction.

### *Shank3*^*−/−*^ mice showed reduced activation of SC to VTA neurons and reduced interneuronal correlations

**Fig. 3:**
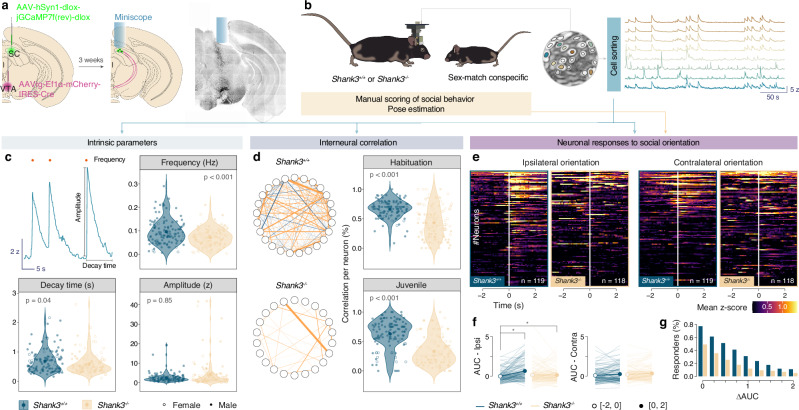
Shank3 KO mice showed reduced activation in neurons controlling orienting response. **a** Schema reporting the viral injections (AAVrg-Ef1α-mCherry-IRES-Cre in the VTA and AAV-hSyn1-dlox-jGCaMP7f(rev)-dlox-WPRE in the SC) and the GRIN lens implantation above the SC. **b** Right panel: schema of the free social interaction test. Left panel: example traces of calcium signals recordings (z-score) from SC-VTA-projecting neurons during the free social interaction test. **c** Intrinsic parameters of the calcium transients recorded in *Shank3*^*+/+*^ (in blue, 119 neurons from 9 mice) and *Shank3*^*−/−*^ (in orange, 118 neurons from 9 mice): frequency (Mann-Whitney test: p-value < 0.001), decay time (Mann-Whitney test: p-value = 0.04), and amplitude (Mann-Whitney test: p-value = 0.85). **d** interneuronal correlation. Left panel: example of estimation of partial correlation networks using EBIC selection (hyperparameter = 0.5). Right panel: violin plots reporting the percentage of significant correlations for a neuron in *Shank3*^*+/+*^ (in blue, 119 neurons from 9 mice) and *Shank3*^*−/−*^ (in orange, 118 neurons from 9 mice) brains during habituation phase (Mann-Whitney test: p-value < 0.001) and interaction with a juvenile (Mann-Whitney test: p-value < 0.001). **e** Neural responses to social orientation. Heatmaps reporting the peri-event time histogram (PETH) of normalized calcium signals (mean z-score) for SC-VTA-projecting neurons in *Shank3*^*+/+*^ (in blue, 119 neurons from 9 mice) and *Shank3*^*−/−*^ (in orange, 118 neurons from 9 mice) centered on ipsi- and contra-recorded orientations. **f** AUC (periods [−2:0] and [0:2]) calculated from the mean z-score reported in **e** per *Shank3*^*+/+*^ and *Shank3*^*−/−*^ neurons (two-way ANOVA with Bonferroni post-hoc comparison. Ipsi: genotype main effect F_(1, 235)_ = 2.580, p  =  0.109, period main effect F_(1, 235)_ = 21.34, p  < 0.001, genotype x period interaction F_(1, 235)_ = 23.19, p  < 0.001. Contra: genotype main effect F_(1, 235)_ = 0.7460, p  =  0.389, period main effect F_(1, 235)_ = 8.489, p  = 0.004, genotype x period interaction F_(1, 235)_ = 0.09, p  = 0.764). **g** Barplot reporting the percentage of responding neurons according to the increase of AUC (AUC^[0; 2]^ - AUC^[−2; 0]^) per *Shank3*^*+/+*^ and *Shank3*^*−/−*^ group. Violin plots are reporting the mean +/− s.e.m. as error bars.

We have previously shown that the activity of the SC to VTA neurons is involved in social orienting behavior in mice [25]. Based on these findings and human samples showing changes in the SC to VTA pathway, we hypothesize that deficits in neuronal activity within the SC neurons projecting to the VTA may contribute to social interaction deficits. To test this hypothesis, we injected a retrograde cre-expressing virus (AAVrg-Ef1α-mCherry-IRES-Cre) in the VTA and cre-dependent AAV encoding GCaMP7s in the SC (AAV-hSyn1-dlox-jGCaMP7f(rev)-dlox-WPRE, Fig. 3a). We implanted a GRIN lens (Inscopix ©) in the SC (Fig. 3a) and we used miniscope to image the calcium transients of SC to VTA neurons during free social interaction with a juvenile sex-matched conspecific (Fig. 3b). We measured the frequency, decay time and amplitude of the calcium transients, the interneuronal correlation as well as calcium transients during specific behavioral bouts. Although we did not observe differences in amplitude between groups, the frequency and decay time of calcium transients of SC to VTA was significantly lower in *Shank3*^*−/−*^ mice (Fig. 3c). Interestingly, the partial correlation networks estimated using the extended Bayesian information criterion (EBIC) revealed that some neurons presented significant correlations in their activity. During social interaction and the preceding habituation phase, *Shank3*^*−/−*^ mice showed significantly lower interneuronal correlations than controls (Fig. 3d), suggesting a suboptimal SC-VTA pathway.

In a previous study, we demonstrated that the SC-VTA pathway is significantly activated during ipsi-recorded orientations towards a conspecific [25]. Consequently, we aligned z-scored signals on ipsilateral orienting events and we replicate our previous findings at the single cell level in *Shank3*^*+/+*^ mice (Fig. 3e, f). Notably, we observed during these events a lower number of activated neurons in *Shank3*^*−/−*^ mice when compared to control (Fig. 3e–g). Interestingly, the activation of these neurons during ipsi-oriented episodes were dependent on their frequency, decay time and amplitude (Supplementary Fig. 1a). On the other hand, contralateral orientations did not provoke an increase of the signal in either *Shank3*^*+/+*^ or *Shank3*^*−/−*^ (Fig. 3e, f) and the neuronal activation was not correlated to the intrinsic parameters (Supplementary Fig. 1b). These data suggest that social orienting deficits may be the consequence of neuronal deficits within the SC to VTA pathway.

### SC to VTA neurons in *Shank3* KO mice presented altered intrinsic properties

**Fig. 4:**
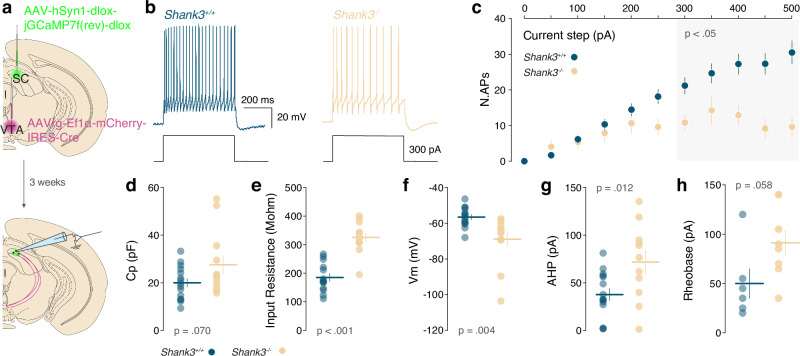
Differences in intrinsic electrophysiological properties in *Shank3* deficient neurons. **a** Schema reporting the viral injections (AAVrg-Ef1α-mCherry-IRES-Cre in the VTA and AAVrg-Ef1α-mCherry- IRES-Cre in the SC). **b** Example traces at 300 pA depolarizing current injection for *Shank3*^*+/+*^ (in blue) and *Shank3*^*−/−*^ (in orange) neurons. **c** Number of action potentials (N.APs) across increasing depolarizing current steps (0–500 pA) for *Shank3*^*+/+*^ (in blue, n = 15, mice = 3) and *Shank3*^*−/−*^ (in orange, n = 11, mice = 3, two-way ANOVA with Bonferroni post-hoc comparison, genotype main effect F_[1,14]_ = 9.49, p  =  0.0081, current steps main effect F_(10, 140)_ = 45.58, p  < 0.001, genotype x current step interaction F_[10,96]_ = 11.57, p  < 0.001). Intrinsic properties of recorded neurons: **d** Capacitance (Cp, unpaired t-test: p = 0.070, *Shank3*^*+/+*^ = 15, *Shank3*^*−/−*^ = 12); **e** Input resistance (unpaired t-test: p < 0.0001, *Shank3*^*+/+*^ = 14, *Shank3*^*−/−*^ = 12); **f** Resting membrane potential (unpaired t-test: p = 0.0037, *Shank3*^*+/+*^ = 15, *Shank3*^*−/−*^ = 12); **g** After-hyperpolarization current (AHP, unpaired t-test: p = 0.012, *Shank3*^*+/+*^ = 15, *Shank3*^*−/−*^ = 12); **h** Rehobase (unpaired t-test: p = 0.058, *Shank3*^*+/+*^ = 6, *Shank3*^*−/−*^ = 8). Each graph reports the mean +/− s.e.m. as error bars.

Using patch-clamp recordings on SC neurons projecting to the VTA, we investigated whether the neuronal activity deficits resulted from alteration in intrinsic neuronal properties. To identify the neurons, we injected a retrograde Cre-expressing virus (AAVrg-Ef1α-mCherry-IRES-Cre) in the VTA and a Cre-dependent reporter in the SC (AAV9-hEF1α-dlox-EGFP-dlox-WPRE, Fig. 4a). After at least 3 weeks, we cut coronal slices and measured whole cell recording in current clamp mode. We observed a decreased number of action potentials (Fig. 4b, c), higher input resistance (Fig. 4e), lower resting membrane potential (Fig. 4f) and a tendency of higher capacitance (Fig. 4d) in slices from *Shank3*^*−/−*^ mice compared to wild type littermates. Interestingly, in the presence of synaptic blockers (picrotoxin and kynurenic acid), while the number of action potentials remained unchanged, we still observed differences in input resistance and rheobase (Supplementary Fig. 2a–g). These results suggest that the deficits observed in neuronal activity arise from a combination of altered intrinsic properties and changes in network activity. The persistence of some alterations in the presence of synaptic blockers indicates that intrinsic neuronal properties play a significant role in the observed phenotype, while the attenuation of excitability changes suggests that network activity also contributes to the overall alterations in SC to VTA neuron function in *Shank3*^*−/−*^ mice.

### SC-VTA impairments are correlated with social behavioral deficits in both rodents and humans

**Fig. 5:**
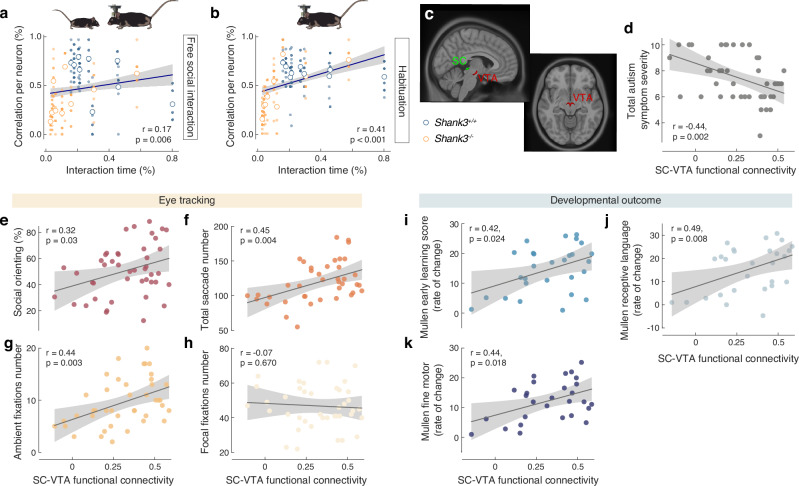
Relation between deficits severity and the SC to VTA pathway in rodents and humans. Relation between percentage of significant correlations for a neuron and interaction time during (**a**) social interaction (during social interaction phase) or (**b**) future interaction (during habituation phase) for *Shank3*^*+/+*^ (in blue, 119 neurons from 9 mice) and *Shank3*^*−/−*^ (in orange, 118 neurons from 9 mice) mice. The mean per each mouse is reported. **c** Selection of the superior colliculus (SC) and ventral tegmental area (VTA) as region of interest (ROI). Panels (**d**–**k**) display scatterplots of SC-VTA functional connectivity (x-axis) against various measures (y-axes) encompassing behavioral symptoms, eye-tracking metrics of visual attention to social stimuli, and developmental outcomes. All panels depict the results of the Pearson correlation, the panel d included Spearman correlation. A median absolute deviation (MAD) threshold of 3 was used to identify and exclude outliers in the correlation analyses. Of note, none of the results changed significance status due to the outlier removal. Panel (**d**) shows the total level of symptoms evaluated by ADOS (n = 45); panel (**e**) percentage of time spent on fixating social stimuli (n = 45); panel (**f**) total number of saccades, n = 40; panel (**g**) number of ambient fixations, n = 41; panel (**h**) number of focal fixations, n = 42. Mullen Early Learning Composite Score rate of change is depicted in (**i**) for the total score, n = 28, (**j**) for the receptive language subdomain, n = 28, and (**k**) for the fine motor subdomain, n = 28. All panels depict the results of the Pearson correlation.

We next verified whether the alterations observed within the SC to VTA pathway both in mice and humans were correlated with the severity of the behavioral symptoms. In mice, the time spent in direct interaction with a social stimulus significantly correlates with the SC-VTA interneuronal correlation (Fig. 5a). Interestingly, we also observed that interneuronal correlation during the habituation phase was positively correlated with the time of future interaction (Fig. 5b). These findings underscore the critical role of SC-VTA pathway in social behavior and propose that the strength of interneuronal correlations might serve as a predictive marker for social exploratory behavior towards other conspecifics.

Based on the pattern of functional connectivity (FC) between the SC and VTA revealed by our multivariate analysis, we further explored how the connectivity between the SC and VTA (ROI to ROI) related to other aspects of behavior in children with ASD (Fig. 5c). Concordant to the results revealed by the PLS-C analyses, SC-VTA functional connectivity pattern showed a negative relationship with the total level of autistic symptoms (Fig. 5d). The SC-VTA FC demonstrated a stronger negative correlation with symptoms in the Social Affect domain (r = −0.39, p = 0.009) compared to the Repetitive and Restricted Behavior (RRB) domain (r = −0.33, p = 0.027), as illustrated in Supplementary Fig. 3a, b. On the contrary, social orienting derived from the visual preference paradigm positively correlated with SC-VTA FC (Fig. 5e). Specifically, more dynamic visual exploration characterized by a higher saccade number (Fig. 5f) and stronger engagement of the ambient fixation mode (Fig. 5g) correlated positively and significantly with SC-VTA FC (r = 0.45, p = 0.004, r = 0.44, p = 0.003 respectively). No significant correlation was found between the number of focal fixations and the SC-VTA FC (Fig. 5h).

To examine the role of the SC-VTA functional connectivity in developmental changes, we used data from any follow-up visits conducted after the initial recording session with the children. In our protocol, these follow-up visits are scheduled at six-month intervals. However, due to the variability in elapsed time between the MRI session and follow-up visits – where some children were reassessed after six months and others also after one year – we devised a ‘rate of change’ score. This score was calculated by dividing the difference between the (furthest) follow-up and baseline assessment results (Mullen Scales of Early Learning - MSEL) by the elapsed time period. This approach allowed us to accommodate the varying follow-up time frames across participants and more accurately assess the contribution of FC connectivity to developmental changes over time. In our study, follow-up data from the MSEL were available for 28 children. Our analysis revealed a generally positive correlation between the developmental rate of change and SC-VTA connectivity (Fig. 5i). This overall effect appeared to be primarily influenced by two developmental domains: receptive language (Fig. 5j) and fine motor skill coordination (Fig. 5k). Both areas necessitate the fine and timely integration of multisensory information. This finding suggests that unaltered SC-VTA connectivity may play a significant role in the developmental acquisitions in these specific cognitive and motor domains.

## Discussion

In this study, we probed the circuit mechanisms underlying social motivation deficits in ASD. Using a translational approach, we revealed reduced connectivity between the SC and VTA, along with alterations in interneuronal correlations of calcium activity and changes in intrinsic cell properties of SC neurons projecting to the VTA. In mice, changes in the activity of SC to VTA pathway are predictive of social interaction, whereas in humans, its functional connectivity pattern inversely correlates with social symptoms. Finally, we found a direct correlation between the developmental trajectory and SC-VTA connectivity.

The *Shank3* mouse model offers a unique opportunity to dissect the neuronal circuits and mechanisms underlying ASD-like behaviors. We used in this study the global De4-22 model which displays aberrant social interaction, altered ultrasonic vocalization and increased repetitive responses [27, 28]. While these mice have been extensively characterized, the origin of social deficits is still largely unknown. Indeed, traditional approaches to studying social interaction, which typically rely on simplistic behavioral assays that score interaction time, fail to capture the behavioral complexity. We focused on social orienting, defined as the ability to attend to salient social stimuli, which is a fundamental behavior across life stages. Our results revealed that mice with an ASD-related mutation show a reduced propensity to orient towards the social stimulus to the conspecific, in contrast to control mice. By exploring these behavioral nuances, we aim to bridge the gap between human and animal models in understanding ASD. Our approach highlights the importance of designing specific behavioral tasks to dissect the multiple alterations associated with ASD, thereby enriching our understanding of the underlying circuit mechanisms.

Pharmacological studies suggest that the disinhibition of the SC increases the responsiveness of DA neurons, and dopamine release in the striatum [24, 52, 53]. Furthermore, it has been recently shown that SC to VTA pathway plays a role in visually evoked innate defensive responses, in social orienting behavior and that disruption of the activity within this pathway leads to deficits in social interaction [25, 26]. Our novel findings indicate that Shank3 knock-out mice present alterations in the activity of SC to VTA pathway. Our study primarily focused on SC neurons projecting to the VTA, revealing altered intrinsic properties and excitability in these cells in *Shank3*^*−/−*^ mice. However, a pertinent question arises regarding the broader impact of Shank3 deletion on SC neuronal excitability. To address this, we conducted additional experiments using glutamate and GABA blockers (picrotoxin and kynurenic acid; see Supplementary Fig. 2). These experiments revealed that changes in input resistance and rheobase persisted even when synaptic transmission was blocked. This suggests that these changes are indeed due to intrinsic properties of the SC neurons. Interestingly, the changes in excitability (number of action potentials) were attenuated when synaptic transmission was blocked, indicating that network activity also contributes to the observed phenotype.

While our study does not directly examine all SC neuronal populations, it is plausible that Shank3 deletion could affect excitability more broadly within the SC, given its role in synaptic function and neuronal excitability in other brain regions. Future studies should investigate excitability changes in diverse SC neuronal populations to provide a comprehensive understanding of how Shank3 deletion impacts this crucial structure. This broader perspective could offer valuable insights into the circuit-wide alterations in ASD and potentially reveal new therapeutic targets.

*Shank3* is a widely expressed scaffolding protein that is enriched in postsynaptic compartments. Interestingly, downregulation of the protein not only causes changes in synaptic properties but also causes channelopathy, with major phenotype associated with impairments in HCN channels [54]. Further studies are needed to determine whether intrinsic properties of SC to VTA neurons are altered due to changes in HCN channels, and whether other ASD-related mouse models exhibit similar alterations.

Our results in animal model, align with human functional connectivity analyses, suggesting that atypical social orienting in ASD may involve early-maturing brain structures, such as the SC, which is implicated in detecting salient environmental cues [19, 55]. This ancient attention pathway is phylogenetically preserved across species to favor fast detection of the relevant signal, such as the threat or conspecific signaling. Human fetal research has shown that this structure matures early, around 20 weeks into the gestation period [45] which predisposes it to relevance detection early on. This is consistent with the early preferential orienting to faces [56], or face-like stimuli [57, 58] seen in human babies. Our data-driven FC analysis showed that the aberrant connectivity of the SC circuitry correlated with the enhanced autistic symptomatology. This finding is in accordance with evidence that associates the SC not only with reflexive behaviors but also with more complex attention and decision-making behaviors, given its extensive input and output connections [59]. We showed that the SC-VTA connectivity was positively related to the social preference as assessed using our eye-tracking paradigm. Additionally, the lower SC-VTA connectivity was associated with a diminished frequency of visual saccadic movements, suggesting a less dynamic visual scanning strategy. Notably, lower SC-VTA connectivity correlated with a decreased reliance on ambient fixation mode (longer saccades shorter fixations). This attentional mode is indicative of a rapid acquisition strategy of the coarse visual information essential for initial scene understanding [60] and is deemed adapted to a paradigm where simple preference trials are shown one after the other [12]. While our study did not include pupillary dilation measures, future research should consider this physiological metric, as it provides direct insights into attentional engagement across both human and animal models.

Our study leverages a mouse model to investigate the circuit mechanisms underlying social orienting deficits. Earlier studies have shown that SC neurons innervate midbrain DA neurons influencing phasic signals that reinforce the selection of movement in response to unexpected biologically relevant events [61]. This process is facilitated by the rapid activation of SC upon visual stimulus detection. Timely encoding and processing of stimuli are essential for survival and adaptation across species. Successful integration of these stimuli must rely on a neurobiological system that is both rapid and capable of handling varying amounts of information pertinent to the specific ethological contexts [62]. For humans, navigating the complexity of the social world is critical for survival, especially considering the extreme and prolonged vulnerability of neonates [9]. Thus, the innate mechanism of social orientation is activated to ensure developmental thriving [3]. Therefore, a reduced sensitivity to social cues early on can significantly impact the trajectory of developmental growth. Using a unique cohort of young children with ASD, this study has taken an important first step in elucidating the role of connectivity between the superior colliculus and the ventral tegmental area (SC-VTA) in mediating the acquisition of new experiences and learning. Children who demonstrated stronger intrinsic connectivity properties within the SC-VTA functional connectivity exhibited greater developmental gains in subsequent months. Our results hint toward the potential of SC-VTA connectivity as a biomarker of outcomes. To further ascertain the role of SC-VTA connectivity as a stratification factor of developmental progress further studies using larger sample sizes are warranted.

## Supplementary information

supplementary information

SUpplementary figure 1

SUpplementary figure 2

SUpplementary figure 3

SUpplementary table

## Data Availability

Mouse: Raw data supporting the findings presented here are available from the corresponding author upon reasonable request. Human: In compliance with data privacy regulations and participant consent, raw MRI data can not be publicly shared. However, preprocessed MRI datasets and derivative results can be made available upon reasonable request.

## Code availability

Custom-made code used in this study is available from the corresponding author upon reasonable request.
