# Supplementary material for: Translational research approach to social orienting deficits in autism: the role of superior colliculus-ventral tegmental pathway
Source: Mol Psychiatry. 2025 Apr 5;30(8):3729–39. doi: 10.1038/s41380-025-02962-w (PMC12240802; doi:10.1038/s41380-025-02962-w)
Supplement: Supplementary file 1 — supplementary information [file 41380_2025_2962_MOESM1_ESM.docx]

**Supplementary information**

**Partial Least Squares Correlation-PLS-C**

To thoroughly explore the connection between behavioral characteristics (such as age and autistic symptoms) and seed-to-whole-brain functional connectivity, we employed behavior partial least squares correlation (PLS-C) (32,33), implemented using the MATLAB-based, publicly available toolbox (https://github.com/MIPLabCH/myPLS). Partial Least Squares Correlation (PLS-C) analyzes the relationship between two matrices, X and Y, which contain measurements from identical observational instances, like scans or participants (s). The X matrix (s x v) represents brain activity, while the Y matrix (s x k) denotes behavioral or design variables. The relationship between a specific column in X (the vth column) and a column in Y (the kth column) is quantified through their dot product. By centering these columns, the dot product reflects their covariance. When columns are normalized—either converted to Z-scores or having their squared values sum to one—the dot product indicates their correlation. Due to the non-directionality of covariance/correlation, X and Y are interchangeable in this analysis, highlighting the extraction of mutual information (33). In our design, we included the SC seed-to-whole-brain FC matrix as X, and age along with measures of autistic symptoms as Y, or design variables. These matrices were used to compute a cross-product matrix (R) as R = Y^T^X. Subsequently, R was decomposed using singular value decomposition (R = UΔV^T^), producing singular vectors U and V, also referred to as saliences. The contribution of brain and behavioral variables to these saliences is expressed through latent components (LCs), which are derived by projecting each subject’s brain imaging data (X) and behavioral data (Y) onto the corresponding salience (V and U, respectively). The generalizability of the results, or their significance, is determined via permutation testing, which involves randomly permuting the rows of X while keeping Y intact, thus establishing a sampling distribution for the singular values across 1000 permutations. The stability of the derived LCs is assessed using a bootstrapping procedure, involving 500 random samples with replacement of the observations in X and Y. This process yields a stability measure for each voxel by dividing the mean of the distribution of brain saliences for each voxel by their respective standard deviation. These bootstrap scores represent an index of the stability of a voxel's response, and can be interpreted akin to z-scores (33). The motion parameters and sex were regressed out from both sides of the PLS design in our study. In the brain maps visualized in this study, bootstrap ratios with an absolute value greater than 3 are depicted, indicating a stable contribution to the LC. Additionally, the design saliences are presented in a bar plot with an error bar indicating their respective bootstrap ratio standard deviation.

**Supplementary analysis of genotype effects and individual mouse robustness on neuronal function parameters**

We performed linear mixed model analyses (lme4 library in R) to assess the effects of genotype on several parameters related to neuronal function.

For frequency, the analysis indicated a moderate positive effect of genotype, with Shank3+/+ mice showing a slight increase compared to Shank3-/- mice (estimate: 0.02346, t = 1.601). Importantly, the low variance attributed to mouse ID (0.0007903) indicates that the frequency changes are consistent across individual mice, reinforcing the reliability of the results.

For the percentage of correlations per neuron, Shank3+/+ mice exhibited a significant increase in the number of correlated neurons compared to Shank3-/- mice during both habituation (Estimate: 0.19327, t = 2.217) and interaction with a juvenile (Estimate: 0.2314, t = 2.239). The low variance associated with the random effects of mouse ID (0.03048 and 0.04322 for the two models, respectively) indicates that these effects are consistent across individual mice, reinforcing the robustness of our findings.

**Supplementary figure 1 - Relation between differences in AUC and the intrinsic properties of a neuron.** Panels in **(a)** report the correlations between the difference in AUC (AUC^[0; 2]^ - AUC^[-2; 0]^) calculated during ipsilateral orientation and neuronal frequency, decay time and amplitude. Panels in **(b)** report the correlations between the difference in AUC (AUC^[0; 2]^ - AUC^[-2; 0]^) calculated during contralateral orientation and neuronal frequency, decay time and amplitude. All panels depict the results of the Pearson correlation and include all *Shank3^+/+^* (in blue, 119 neurons from 9 mice) and *Shank3^-/-^* (in orange, 118 neurons from 9 mice) neurons.

**Supplementary figure 2 - Differences in intrinsic electrophysiological properties in *Shank3* deficient neurons in presence of blockers. (a)** Schema reporting the viral injections (AAVrg-Ef1α-mCherry-IRES-Cre in the VTA and AAVrg-Ef1α-mCherry- IRES-Cre in the SC) and ex vivo recording in presence of in presence of Picrotoxin (100 µM) and Kynurenic acid (3 mM)**. (b)** Number of action potentials (N.APs) across increasing depolarizing current steps (0-500 pA) for *Shank3^+/+^* (in blue, n = 8, mice = 3) and *Shank3^-/-^* (in orange, n = 12, mice = 3) in presence of blockers (two-way ANOVA, genotype main effect F_(1, 18)_ = 3.488, p = 0.08, current steps main effect F_(10, 180)_ = 88.66, p < 0.001, genotype x current step interaction F_(10, 180)_ = 1.04, p = 0.41). Intrinsic properties of recorded neurons: **(c)** Capacitance (Cp, unpaired t-test: p < 0.001, *Shank3^+/+^* = 8, *Shank3^-/-^* = 8); **(d)** Input resistance (unpaired t-test: p < 0.001, *Shank3^+/+^* = 8, *Shank3^-/-^* = 12); **(e)** Resting membrane potential (unpaired t-test: p = 0.014, *Shank3^+/+^* = 8, *Shank3^-/-^* = 12); **(f)** After-hyperpolarization current (AHP, unpaired t-test: p = 0.176, *Shank3^+/+^* = 8, *Shank3^-/-^* = 12); **(g)** Rehobase (unpaired t-test: p = 0.041, n *Shank3^+/+^* = 8, n *Shank3^-/-^* = 12). Each graph reports the mean +/- s.e.m. as error bars.

**Supplementary figure 3 - Relation between deficits severity and the SC to VTA pathway.** Panels **(a-b)** display scatterplots of SC-VTA functional connectivity (x-axis) against social affect symptom severity (a, n = 45) or restricted and repetitive behaviors (RRB) symptom severity (b, n = 45). All panels depict the results of the Pearson correlation.
