# Supplementary figures and images for: Translational research approach to social orienting deficits in autism: the role of superior colliculus-ventral tegmental pathway
Source: Mol Psychiatry. 2025 Apr 5;30(8):3729–39. doi: 10.1038/s41380-025-02962-w (PMC12240802; doi:10.1038/s41380-025-02962-w)

**a**

Ipsilateral orientation

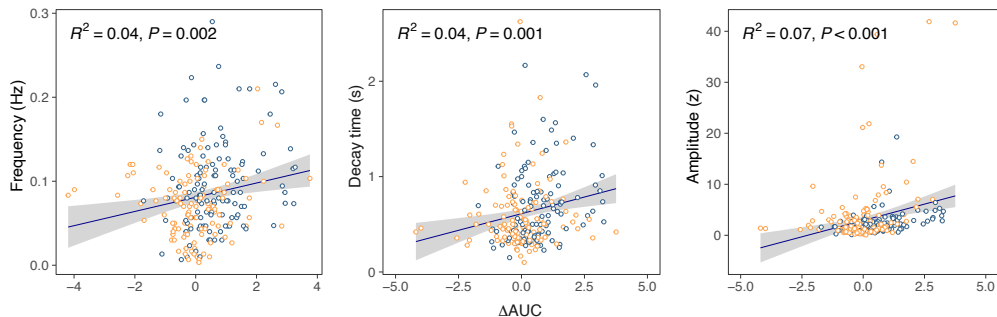**b**

Contralateral orientation

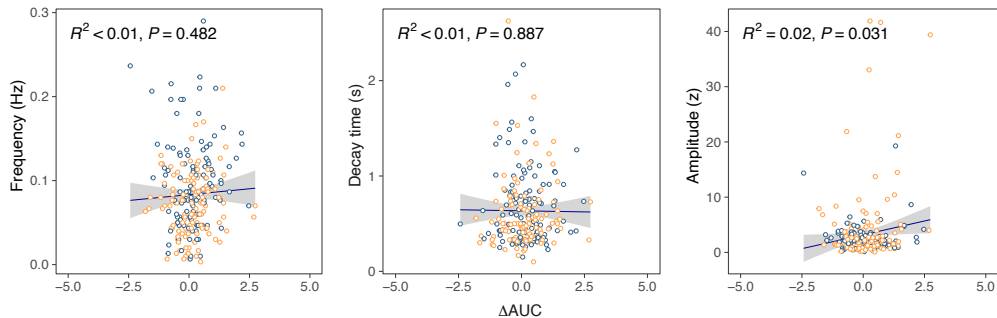● *Shank3*<sup>+/+</sup> ● *Shank3*<sup>-/-</sup>

Supplement: Supplementary file 2 — SUpplementary figure 1 [file 41380_2025_2962_MOESM2_ESM.pdf]

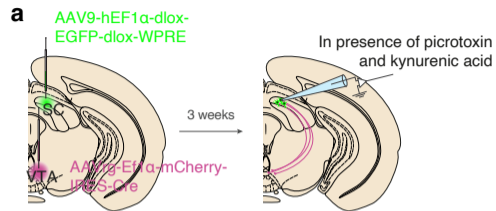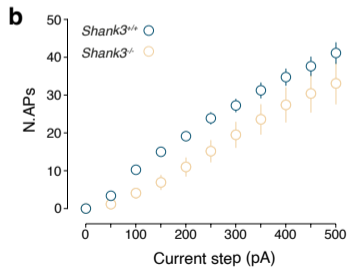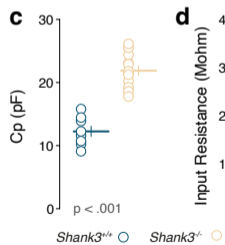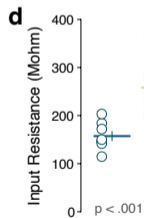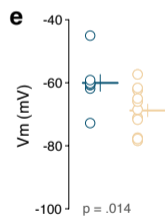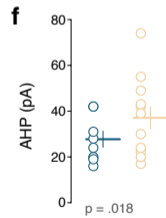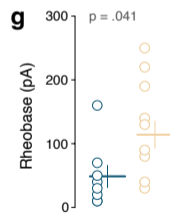

Supplement: Supplementary file 3 — SUpplementary figure 2 [file 41380_2025_2962_MOESM3_ESM.pdf]

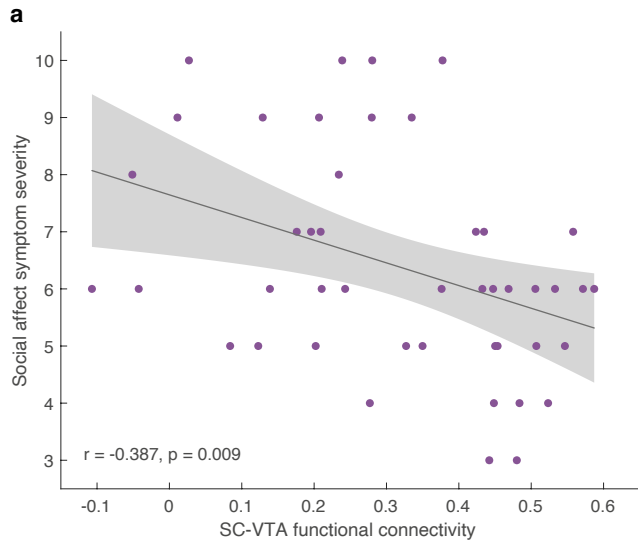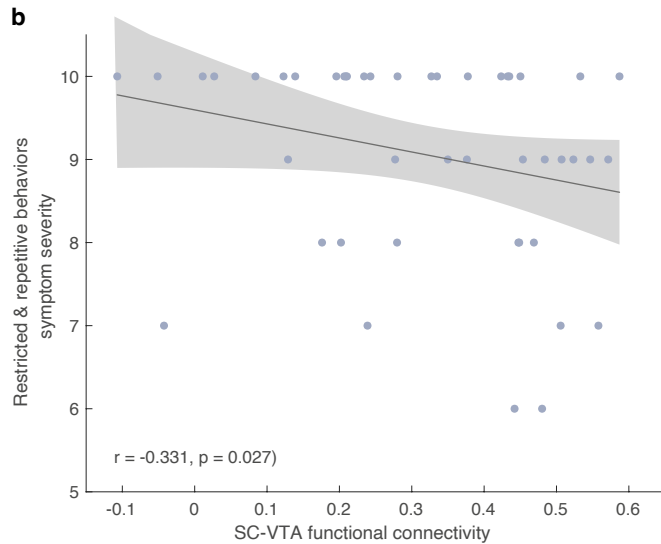

Supplement: Supplementary file 4 — SUpplementary figure 3 [file 41380_2025_2962_MOESM4_ESM.pdf]
